# Multicenter Evaluation of the First Validated German-Language Fatigue Questionnaire for Patients with Chronic Inflammatory Bowel Diseases

**DOI:** 10.3390/jcm14113618

**Published:** 2025-05-22

**Authors:** Magnus Müller, Franziska Schulz, Vidan Tadic, Anna Muzalyova, Johanna Classen, Ulrike Denzer, Irina Blumenstein, Elisabeth Schnoy

**Affiliations:** 1 Internal Medicine III, Department of Gastroenterology, University Hospital Augsburg, 86156 Augsburg, Germany; vidan.tadic@uk-augsburg.de (V.T.);; 2 Medical Clinic 1, Frankfurt University Hospital, Goethe University Frankfurt, 60596 Frankfurt, Germanyirina.blumenstein@unimedizin-ffm.de (I.B.); 3 Fraunhofer Institute for Translational Medicine and Pharmacology ITMP, 60596 Frankfurt, Germany; 4 Institute for Digital Medicine, University Hospital of Augsburg, 86156 Augsburg, Germany; anna.muzalyova@uk-augsburg.de; 5 Division of Interdisciplinary Endoscopy, University Hospital of Giessen and Marburg Campus Marburg Clinic for Gastroenterology Endocrinology Metabolism and Clinical Infectiology, 35043 Marburg, Germany; ulrike.denzer@uk-gm.de

**Keywords:** fatigue, inflammatory bowel disease, patient-reported outcomes, symptom burden, quality of life

## Abstract

**Background**: Patients with inflammatory bowel disease (IBD) often suffer from extra-intestinal manifestations in addition to intestinal symptoms. One of these is fatigue. Fatigue is described as persistent tiredness with episodes of sudden energy loss, which cannot be relieved by rest or sleep and has a huge impact on quality of life. The aim of this study is to identify possible risk and influencing factors for the development of fatigue in IBD. **Methods**: For this purpose, a questionnaire survey was conducted at two German university outpatient clinics for IBD (n = 164). Based on this, the frequency and impact of fatigue on daily life was assessed and analyzed in relation to various health parameters such as patient gender, age, disease activity, and laboratory parameters. **Results**: Of the 164 patients, 86 were men (52.4%) and 78 were women (47.6%). A total of 75 (45.7%) patients had ulcerative colitis, 84 (51.2%) suffered from Crohn’s disease, and 5 (3.0%) had IBD-unclassified. A total of 17 out of the 164 (10.4%) patients denied that fatigue had affected their daily activities in the past two weeks. None of the examined health parameters had a significant impact on fatigue. **Conclusions**: Fatigue is a common syndrome in IBD patients and affects their daily activities and quality of life. The results of the present study emphasize the need for further research for a better scientific understanding of fatigue in IBD.

## 1. Introduction

Chronic inflammatory bowel diseases (IBDs) such as Crohn’s disease (CD) and ulcerative colitis (UC) are conditions characterized by inflammation of the gastrointestinal tract that occurs in episodes. There are 6–8 million IBD sufferers worldwide, and the number of those affected is rising steadily. In addition to intestinal symptoms such as abdominal pain and diarrhea, patients also suffer from extra-intestinal manifestations. These can manifest themselves in the form of skin and joint complaints. Furthermore, there is an increased prevalence of accompanying mental illnesses such as depression and anxiety disorders, as well as fatigue syndrome [1].

Fatigue refers to persistent tiredness associated with episodes of sudden, overwhelming loss of energy or exhaustion, which cannot be relieved by rest or sleep [2].

Fatigue is persistent tiredness associated with a sudden, overwhelming loss of energy or exhaustion that cannot be relieved by rest or sleep. Persistent fatigue can lead to significant limitations in the patient’s quality of life. In severe cases, it can result in the loss of productivity and sickness absence at work, as well as high costs for the healthcare system [3,4].

According to Lönnfors et al., fatigue is even the primary cause of work disability in IBD patients, preceding gastrointestinal symptoms in a European cohort [3,4].

The causes of fatigue are diverse and not fully understood, ranging from nutrient deficiencies and psychosocial factors to disease activity. A standardized treatment is currently lacking, even though fatigue affects the majority of IBD patients [2,5].

There are various levels at which fatigue could arise. Differences in the levels of TNFα and IL-6, as well as distinct leukocyte profiles, have been identified in patients with varying degrees of fatigue, suggesting that one level of fatigue may be immunologically mediated. Some studies have shown a correlation between the activity of IBD and fatigue in terms of its frequency and severity [6,7].

Psychosocial factors also seem to play a role in the pathogenesis of fatigue. For example, depression and a low quality of life are also associated with fatigue. Furthermore, sleep quality appears to play a crucial role. A study showed that sleep disturbance increased the risk of fatigue by four times. A lower sleep quality is more common in patients with inflammatory bowel disease than in the general population [8,9,10].

Moreover, some studies have shown a correlation between certain nutrient deficiencies and fatigue in IBD, while others have not, making the study results unclear. These include vitamins such as vitamin D, B12, folic acid, as well as hypophosphatemia. Iron deficiency and the resulting anemia can also contribute to fatigue in IBD [11,12,13,14,15,16].

Furthermore, the altered gut microbiome in IBD seems to play a role. The bidirectional communication between the microbiome and the brain, referred to as the gut–brain axis, could play a role in the pathogenesis of fatigue [17,18].

Although the pathophysiology of fatigue has been better understood through previous studies, a unified diagnostic and therapeutic approach is still lacking. The German guidelines for fatigue in patients with Crohn’s disease include, in addition to the regular assessment of fatigue and optimal pharmacological treatment of IBD, the management of comorbidities. This includes the exclusion of vitamin and nutrient deficiencies as well as the exclusion of underlying psychiatric disorders. While short-term thiamine supplementation seems to have a positive effect on fatigue symptoms, the long-term effect is as unclear as the role of probiotics in altering the gut–brain axis. The evidence regarding the impact of physical exercise on fatigue is inconclusive. A multidisciplinary approach seems necessary [5,19,20,21,22].

Since the pathophysiology is complex and not fully understood, and there is no standardized approach regarding diagnosis and treatment, fatigue remains an underaddressed issue in IBD patients [23].

Due to the high prevalence of fatigue and its associated impact on quality of life in patients with IBD, coupled with the lack of standardized treatment approaches, there is a clear need for further research to better understand this complex issue.

There are several fatigue scales that were not originally developed specifically for IBD patients but have been used in studies involving them, such as the FQ and the MFI-20 [24,25,26].

Specifically for the assessment of fatigue in IBD patients, the IBD-F was developed by Czuber-Dochan et al. in 2014. The IBD-F shows a good correlation with other fatigue scales, such as the MFI-20. Nevertheless, there is no unified recommendation regarding which questionnaire should be used to assess fatigue [5,8,27].

Scholz et al. translated and validated the IBD-F into German, making it the first validated German-language questionnaire for assessing fatigue in IBD patients [28].

## 2. Materials and Methods

### 2.1. Patient Demographics and Study Design

For the present study, the IBD-Fatigue questionnaire was used to assess the prevalence and impact of fatigue in IBD patients. A total of 164 patients with IBD were interviewed at two study centers, the University Hospital Augsburg and the University Hospital Frankfurt. The questionnaire survey took place during consultations (questionnaire can be found in Table S1).

This study was approved by the ethics committee of the University Hospital Regensburg, Germany (Ethical committee approval dated 26 January 2024, vote number 24-3617-101). Each participant signed an informed consent form. Data were analyzed anonymously.

In addition to questions on demographics, medical history, and disease status including clinical activity, the following parameters were recorded for disease activity in IBD in the questionnaire for each individual subject: histology, imaging and/or fecal calprotectin (fCal), and C-reactive protein (CRP) [29]. In addition, the current therapy; vitamin D3; vitamin B12; folic acid; iron status including ferritin, transferrin, and transferrin saturation; hemoglobin; and albumin were recorded.

In the context of this non-interventional study, no additional examinations were performed on the patients that were not part of routine clinical care. Therefore, the collection of various parameters, such as disease activity or different laboratory values, was not available for all patients.

The IBD-F was divided into different parts. The first part addressed the severity and frequency of fatigue through 5 questions on a 5-point Likert scale, and the second area covered the impact on daily life over the past two weeks with 30 questions on a 5-point Likert scale. Higher scores on the Likert scale indicated a higher level of fatigue in terms of frequency and daily impact. The provided point values on the Likert scale were added up, resulting in a sum for each area that was intended to reflect the burden of fatigue (SCORE 1 and SCORE 2). The 3 open-ended questions inquired about the causes of fatigue and potential solutions from the patients’ perspective. Finally, the questionnaire asked about the overall duration of fatigue and whether it was constant or fluctuated.

Since there was no uniform threshold for the presence of fatigue or severe fatigue in the IBD-F, the assessment was performed on an interval scale [30,31].

### 2.2. Statistics

Categorical variables were reported as absolute numbers and percentages. Interval-scaled variables were presented as means with the standard deviation (SD), as appropriate. Comparisons of categorical variables were performed using the Chi-squared test or Fisher’s exact test, with the latter applied when at least one cell contained fewer than five observations. A comparison of interval-scaled variables was performed using the Mann–Whitney-U test. The significance level was set to α = 0.05.

Data management and calculation of descriptive statistics were performed using Excel (Microsoft Corporation, Redmond, WA, USA). Inference-statistical analysis was performed in SPSS 28.0. (Statistical Package for the Social Sciences, Excel (Microsoft Corporation, Redmond, WA, USA)).

## 3. Results

### 3.1. Population

A total of 164 patients were included in the study. Of these, 86 (52.4%) were male and 78 (47.6%) were female. The mean age was 42.7 (SD 13.7) years, with a median of 40.5 years. In our cohort, 84 (51.2%) patients were suffering from Crohn’s disease, 75 (45.7%) had ulcerative colitis, and 5 (3.0%) had IBD-unclassified. For 163 (99.4%) patients, the current therapy could be determined, with the most common therapy being TNF-alpha antagonists (n = 50), followed by vedolizumab (n = 37) and ustekinumab (n = 23). Eight patients were currently without therapy (4.9%). Current disease activity was assessed for 104 patients (63.4%) based on histology, while no current data were available for 60 patients (36.6%). Of these 104 patients, 22 had high inflammatory activity (21.2%), while 24 patients showed no inflammatory activity (23.1%).

Table 1 provides an overview of the collected patient data.

### 3.2. Responses in the IBD-F

As described, the IBD-F is divided into three sections. The meaning of SCORE 1 and SCORE 2 is described in the Methods section.

**Figure 1:**
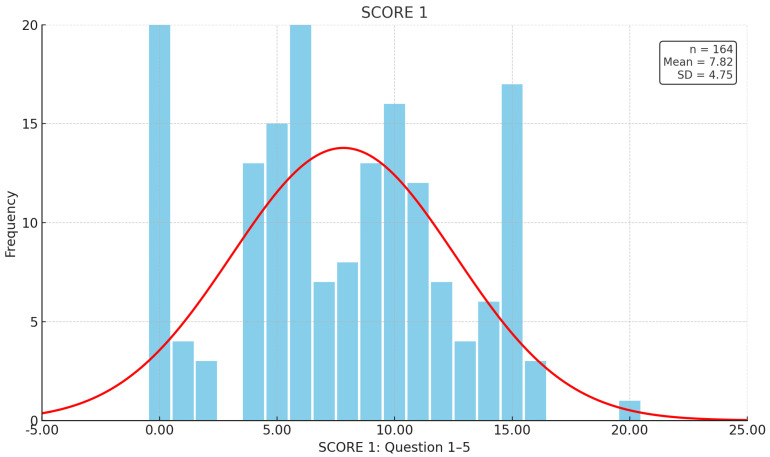
Responses in the IBD-F. The *x*-axis represents the sum of the answers from questions 1–5, with each question scored from 0 to 4. The *y*-axis shows the number of people who reported each sum.

For the first section of the questionnaire (Figure 1) with the questions on the severity and frequency of fatigue, we recorded a mean SCORE 1 of 7.82 points (SD 4.75), with 12.2% answering all five questions with 0 points (n = 20), while only one patient gave the maximum score of 20 points. Together with those who reported never experiencing fatigue in the past 2 weeks, the most common response was 6 points (n = 20). Thus, of the 164 patients surveyed, 144 (87.8%) reported experiencing fatigue at least once in the last two weeks (SCORE 1 > 0).

**Figure 2:**
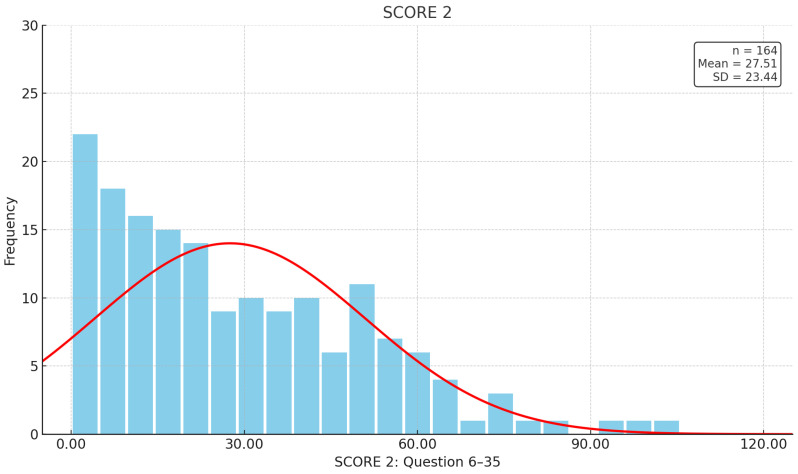
Responses in the IBD-F. The *x*-axis represents the sum of answers from questions 6–35, each scored from 0 to 4, with seven bars corresponding to 30 points. The *y*-axis shows the number of people reporting each sum.

For the second part of the questionnaire (Figure 2), which assessed the impact on daily activities and consisted of 30 questions, the mean SCORE 2 was 27.51 across the cumulative 30 questions (SD 23.445). The most frequent response was 0 points, with 17 patients giving 0 points for all 30 questions. The highest score was 104 points across the 30 questions (n = 1). Thus, 147 (89.6%) patients indicated that fatigue sometimes prevented them from completing at least one daily activity (SCORE 2 > 0).

The average duration of fatigue was reported as 67.45 months (SD 69.3), with a median of 48 months, though only 122 patients answered this question. For 67.9%, fatigue was reported as a fluctuating symptom (n = 91), while 32.1% of patients stated that fatigue was a constant problem.

In the three open-ended questions, which asked patients about what they believed to be the main causes of fatigue and possible solutions, a wide range of responses were given. Frequently mentioned causes included a high burden of IBD symptoms with sleep disturbances, nutrient deficiencies, and medication side effects. Psychosocial factors such as anxiety and stress, whether familial or work-related, were also noted. Just as variable as the suspected causes were the responses regarding coping with fatigue. In addition to social support from family, patients also mentioned regular physical activity and consciously ensuring rest as ways to manage fatigue.

### 3.3. Comparison of IBD-F Responses with the Patient Population

No consistent significant correlation was found between the responses on the IBD-F and the parameters we recorded. A significant association could not be shown for gender, for SCORE 1 (*p* = 0.94), for SCORE 2 (*p* = 0.82), nor for the type of IBD (SCORE 1 *p* = 0.85, SCORE 2 *p* = 0.98). There was also no significant correlation with the currently administered therapy (SCORE 1 *p* = 0.21, SCORE 2 *p* = 0.39). For the laboratory values examined, no significant correlation was found for SCORE 1 or for SCORE 2, except for vitamin D for SCORE 1 (*p* = 0.017).

## 4. Discussion

The frequency of fatigue in studies varies widely, with our data being on the higher end of the spectrum. In a study conducted in France and Belgium by Amiot et al., the frequency ranged between 45% and 65%, depending on disease activity, while other studies reported frequencies over 70% in patients with high IBD activity. It is worth mentioning that in our study, no cutoff was established to define what constitutes fatigue. In other studies, however, a cutoff was set to identify significant fatigue. For example, Varbobitis et al. used a SCORE > 7.5 as the threshold, while Bager et al. compared fatigue in IBD patients to the general population, defining fatigue only when the score exceeded the 95th percentile of the general population. Since there is no unified standard for this cutoff, we decided not to apply one, which helps explain why the prevalence appeared higher in our cohort [5,30,32,33].

In our study, no statistically significant correlation was found for any of the examined parameters, including gender, age, type of IBD, current therapy, and various laboratory parameters such as iron status, vitamins, hemoglobin, and CRP. The only significant correlation observed was an increased vitamin D level, which was associated with a higher fatigue score. The role of vitamin D in relation to fatigue remains unclear. One study by Frigstad et al., for instance, found no significant correlation between vitamin D levels and fatigue in IBD patients [14]. In contrast, a meta-analysis in patients with multiple sclerosis showed that vitamin D supplementation can reduce the burden of fatigue [34]. As mentioned, the vitamin D level was only recorded for 119 of the 164 patients. Therefore, the missing correlation could be due to the incompleteness of the data.

Some of the investigated parameters are well established in the literature, such as the increased burden of women in association with disease activity [2,3,5]. For example, Amiot et al. showed that the female gender is a risk factor for fatigue, with an odds ratio (OR) of 1.48 for general fatigue and 1.37 for severe fatigue, compared to the male gender [32]. This is consistent with data from a Danish study by Bager et al., which included a healthy control population from Denmark, where the fatigue scores in IBD-F were also higher for women than for men, both for SCORE 1 (7.2 vs. 6.6; *p* < 0.001) and SCORE 2 (17.0 vs. 13.5; *p* < 0.001) [35].

Singh et al. showed the dependence of fatigue on disease activity. They reported that 15% of patients without activity still experienced fatigue, while in patients with fatigue, depending on whether they suffered from Crohn’s disease or ulcerative colitis, the prevalence ranged between 38% and 63%, with a higher burden in patients with CD [7]. The increased risk of fatigue in women, the association with active inflammation in IBD, and the more frequent fatigue in Crohn’s disease compared to ulcerative colitis were also described by Cohen et al. [36].

There are several other parameters that may be associated with fatigue in IBD patients, such as hypophosphatemia or a thiamine deficiency. These parameters were not routinely assessed and were therefore not included in our study cohort. Zoller et al. demonstrated that iron substitution for iron deficiency anemia in IBD patients generally improved fatigue levels. However, the improvement was dependent on the specific iron formulation used and the resulting hypophosphatemia. In the TARIF study by Bager et al., a short-term benefit of high-dose thiamine supplementation was observed, while the fatigue level did not differ after 6 months [16,20,37]. Another study showed that infliximab and adalimumab levels below or above therapeutic thresholds did not affect fatigue severity [38].

As mentioned in the introduction, fatigue remains a common and serious issue in patients with IBD. It has been described that fatigue not only has a significant personal impact on the daily lives of affected individuals but also has a relevant societal impact, as patients experience illness more frequently, leading to high healthcare costs for the public.

The frequency and impact on daily life were documented in our cohort from two German university outpatient clinics using the German IBD-F. Although, as described, more knowledge about pathophysiology has been gained in recent years, there is still no standardized diagnostic and treatment pathway to systematically assist affected individuals. However, it is worth noting the undoubtedly growing research interest in fatigue in IBD patients. A search of the PubMed database using the keywords ‘fatigue’ and ‘inflammatory bowel disease’ between 2005 and 2014 yielded only 198 results, whereas between 2015 and 2024, a total of 680 results were found.

We showed that the German IBD-F was easily applicable in daily routine use while showing that fatigue has a huge impact on almost all patients included in our study. Using the German IBD-F is helpful to screen for patients with IBD and fatigue and to analyze the impact of fatigue on the individual patient.

### Limitations of the Study

Incomplete data were available for some subjects in the previous study, as this non-interventional study only included data that were already available in the patient files. Furthermore, it was not possible to determine a statistical cut-off value that defined a distinction between fatigue and severe fatigue. Whether the definition of a cut-off would have resulted in statistically significant correlations for the parameters we investigated was not examined and therefore remains unclear. The present study lacked the inclusion of psychological factors such as depression, anxiety, or sleep quality in the context of comorbidities in IBD. As a result, it was not possible to assess the influence of psychological stress on the development of fatigue. With this study, we show that fatigue is a common and everyday problem for IBD patients in German university outpatient clinics.

## 5. Conclusions

The results of the study show that patients attending German university outpatient clinics for IBD often suffer from fatigue, which significantly impacts their daily lives. In the present study, no significant risk factor for the presence of fatigue could be identified. Further research is needed to determine the causes of fatigue in IBD.

## Figures and Tables

**Table 1:** Overview of the collected patient data including the current therapies (including a pie chart of the therapies) and disease activity. n indicates the number of individuals for whom data are available for the respective characteristic.

Patient-related data (total n = 164)	n	Gender		
	86	Male		52.4%
	78	Female		47.6%
Current therapy (n = 163)	n	Therapy		
	50	TNF-alpha antagonist		30.7%
	37	Vedolizumab		22.7%
	23	Ustekinumab		14.1%
	11	Risankizumab		6.7%
	3	Steroids		1.8%
	3	Azathioprine		1.8%
	28	Other		17.2%
	8	No current therapy		4.9%
Current disease activity (n = 104) based on histology	n	Disease		
	24	None		23.1%
	30	Light		28.8%
	28	Moderate		26.9%
	22	High		21.2%
Laboratory values	n	Laboratory values	Average values	Reference range
	119	Vitamin D	29.6 ng/mL (SD 11.5)	20–70 ng/mL
	145	Vitamin B12	494.5 pg/mL (SD 287.8)	191–663 pg/mL
	82	Folic acid	8.6 ng/mL (SD 5.0)	3.9–26.8 ng/mL
	139	Ferritin	133.7 ng/mL (SD 185.0)	30–400 ng/mL
	121	Transferrin	257.9 mg/dL (SD 34.8)	200–360 mg/dL
	121	Transferrin saturation	24.6% (SD 10.0)	16–45%
	161	Hb	13.9 g/dL (SD 1.7)	14–18 g/dL
	135	Albumin	4.4 g/dL (SD 0.3)	3.5–5.2 g/dL
	159	CRP	0.5 mg/dL (SD 1.2)	<0.5 mg/dL
	75	fCalprotectin	263.3 µg/g (SD 304.2)	<50 µg/g
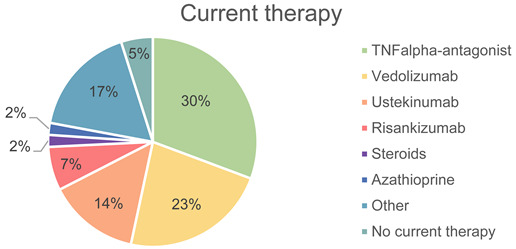				

## Data Availability

The data that support the findings of this study are available from the corresponding author upon reasonable request. The data were collected anonymously, ensuring no restrictions on personal information.

## Supplementary Materials

The following supporting information can be downloaded at: https://www.mdpi.com/article/10.3390/jcm14113618/s1, Table S1: Fatigue questionnaire.
