# Supplementary material for: Multicenter Evaluation of the First Validated German-Language Fatigue Questionnaire for Patients with Chronic Inflammatory Bowel Diseases
Source: J Clin Med. 2025 May 22;14(11):3618. doi: 10.3390/jcm14113618 (PMC12155584; doi:10.3390/jcm14113618)
Supplement: Supplementary file 1 [file jcm-14-03618-s001.zip › jcm-3613945-Supplementary.pdf]

Table S1: Fatigue questionnaire

SECTION I - Fatigue Assessment Scale

	0 = no fatigue				4 = Severe fatigue
1. What is your fatigue level right NOW	0	1	2	3	4
2. What was your HIGHEST fatigue level in the past two weeks	0	1	2	3	4
3. What was your LOWEST fatigue level in the past two weeks	0	1	2	3	4
4. What was your AVERAGE fatigue level in the past two weeks	0	1	2	3	4

	None of the time	Some of the time	Often	Most of the time	All the time
5. How much of your waking time have you felt fatigued in the past two weeks	0	1	2	3	4

SECTION II – IBD-Fatigue Impact on Daily Activities Scale

	None of the time	Some of the time	Often	Most of the time	All the time	Not applicable
1. I had to nap during the day because of fatigue	0	1	2	3	4	
2. Fatigue stopped me from going out to social events	0	1	2	3	4	
3. I was not able to go to work or college because of fatigue	0	1	2	3	4	N/A
4. My performance at work or education was affected by fatigue	0	1	2	3	4	N/A
5. I had problems concentrating because of fatigue	0	1	2	3	4	
6. I had difficulty motivating myself because of fatigue	0	1	2	3	4	
7. I could not wash and dress myself because of fatigue	0	1	2	3	4	
8. I had difficulty with walking because of fatigue	0	1	2	3	4	
9. I was unable to drive as much as I need to because of fatigue	0	1	2	3	4	N/A
10. I was not able to do as much physical exercise as I wanted to because of fatigue	0	1	2	3	4	
11. I had difficulty continuing with my hobbies/interests because of fatigue	0	1	2	3	4	

	None of the time	Some of the time	Often	Most of the time	All the time	Not applicable
**12. My emotional relationship with my partner was affected by fatigue**	0	1	2	3	4	N/A
**13. My sexual relationship with my partner was affected by fatigue**	0	1	2	3	4	N/A
**14. My relationship with my children was affected by fatigue**	0	1	2	3	4	N/A
**15. I was low in mood because of fatigue**	0	1	2	3	4	
**16. I felt isolated because of fatigue**	0	1	2	3	4	
**17. My memory was affected because of fatigue**	0	1	2	3	4	
**18. I made mistakes because of fatigue**	0	1	2	3	4	
**19. Fatigue made me irritable**	0	1	2	3	4	
**20. Fatigue made me frustrated**	0	1	2	3	4	
**21. I got words mixed up because of fatigue**	0	1	2	3	4	
**22. Fatigue stopped me from enjoying life**	0	1	2	3	4	
**23. Fatigue stopped me from having a fulfilling life**	0	1	2	3	4	
**24. My self-esteem was affected by fatigue**	0	1	2	3	4	
**25. Fatigue affected my confidence**	0	1	2	3	4	
**26. Fatigue made me feel unhappy**	0	1	2	3	4	
**27. I had difficulties sleeping at night because of fatigue**	0	1	2	3	4	
**28. Fatigue affected my ability to do all my normal household activities**	0	1	2	3	4	
**29. I had to ask others for help because of fatigue**	0	1	2	3	4	
**30. Quality of my life was affected by fatigue**	0	1	2	3	4	

SECTION III – Additional Questions about your Fatigue

1. What do you think is the main cause of your fatigue apart from IBD?
2. What do you think are the other causes of your fatigue?
3. Have you found anything that helps with your fatigue?
4. How long have you experienced fatigue? ……..years ……….. months
5. During this time has your fatigue been: a) Constant b) Intermittent
